# Burden of Acute Care Surgery and Trauma-Related Mortality in the US State Prisons

**DOI:** 10.1001/jamanetworkopen.2025.31785

**Published:** 2025-09-12

**Authors:** Totadri Dhimal, Paula Cupertino, Owen Tolbert, Yusheng Jia, Bailey Hilty Chu, Raquel Arias-Camison, Camila Lage, Daniela Matute, Yue Li, Fergal Fleming, Anthony Loria

**Affiliations:** 1 Surgical Health Outcomes & Research Enterprise (SHORE), Department of Surgery, University of Rochester Medical Center, Rochester, New York; 2 Division of Health Policy and Outcomes Research, Department of Public Health Sciences, University of Rochester Medical Center, Rochester, New York

## Abstract

This cohort study of US prisons examines changes in mortality rates related to trauma and acute care surgery, including by interstate variation.

## Introduction

The US Department of Corrections (DOC) spends over $8.1 billion annually on health care for nearly 2 million incarcerated individuals.^1^ Despite this substantial investment, the surgical care of incarcerated individuals is poorly understood.^2,3^ While some studies suggest low postoperative mortality, a single-county postmortem analysis found that nearly 20% of in-prison deaths were attributable to trauma or acute care surgical (ACS) conditions.^2,4^ Importantly, among those with noncirrhotic surgical conditions, nearly two-thirds died without operative intervention.^2,4^ Given these possible disparities, our study aims to examine trends in trauma- and ACS-related mortality over time and assess interstate variation, guided by the disease classification framework described by Busko et al.^4^

## Methods

All-cause mortality data collected by UCLA Law’s Behind the Bars Data Project were reviewed and classified by 2 physicians and a medical student (T.D., A.L., and O.T.) using a framework developed in Miami-Dade County, Florida.^4,5^ Demographics, including age, sex, race and ethnicity, and cause of death were extracted. Race, ethnicity, and sex were reported by the DOC and included the following categories: American Indian, Native Hawaiian, or Pacific Islander; Black; Hispanic; White; and unknown. Five states did not report individual sex; 6 states did not report individual race or ethnicity. Mortality rates were adjusted for the state-to-national incarceration rate ratio. χ^2^ and *t* tests were used as appropriate to test for independence; all tests were 2-sided, and *P* < .05 was considered significant. This study was approved by the Rochester Medical Center institutional review board with an exemption for informed consent because data were from deceased individuals. The study follows the Strengthening the Reporting of Observational Studies in Epidemiology (STROBE) reporting guideline for cohort studies.

## Results

Between 2015 and 2020, 8950 deaths occurred in prison systems across 15 different states. Of these, 710 (7.9%) were attributed to traumatic causes and 831 (9.3%) were due to ACS pathologies. Excluding the 5 states that did not report individual sex, 6807 decedents (96.3%) were male; excluding the 6 states not reporting race and ethnicity, 2468 decedents (34.5%) were Black, 924 Hispanic (12.9%), and 3560 White (49.7%) (Table 1). Individuals who died from trauma were younger (median [IQR] age, 39 [30-49] years) compared with those with ACS-related deaths (median [IQR], 58 [52-64] years) or other causes (median [IQR], 61 [53-69] years).

**Table 1.** Demographic and Cause of Death Data

Variables	Deaths, No. (%)			*P* value
	Acute surgical pathology (n = 831)	Trauma (n = 710)	Other (n = 7409)	
Age, median (IQR), y	58 (52-64)	39 (30-49)	61 (53-69)	<.001
Sex				
Female	33 (4.0)	23 (3.2)	209 (2.8)	.02
Male	660 (79.4)	557 (78.5)	5674 (76.6)	
Unknown	138 (16.6)	130 (18.3)	1526 (20.6)	
Race and ethnicity				
American Indian, Native Hawaiian, or Pacific Islander	12 (1.4)	7 (1.0)	49 (0.7)	<.001
Black	147 (17.7)	164 (23.1)	2157 (29.1)	
Hispanic	144 (17.3)	88 (12.4)	692 (9.3)	
White	372 (44.8)	303 (42.7)	2885 (38.9)	
Unknown	156 (18.8)	148 (20.8)	1626 (22.0)	
Location of death				
En route to the medical facility	5 (0.6)	5 (0.7)	13 (0.2)	<.001
Medical facility	367 (44.2)	138 (19.4)	2540 (34.3)	
Prison	131 (15.8)	181 (25.5)	1131 (15.3)	
Unknown	328 (39.5)	386 (54.4)	3725 (50.3)	

Asphyxiation was the leading cause of traumatic death (451 [63.5%]), followed by blunt trauma (98 [13.8%]), penetrating trauma (38 [5.4%]), and unspecified traumatic mechanisms (123 [17.3%]) (Table 1). Suicide accounted for 531 traumatic deaths (74.8%), with state-level proportions ranging from 46.2% to 100% of all traumatic deaths.

Among ACS-related deaths, cirrhosis accounted for 571 (68.7%), followed by hemorrhage (gastrointestinal, cavitary, and aneurysmal) (132 [15.9%]), perforated viscus (26 [3.1%]), and other causes (Table 2). Of individuals with reported locations of deaths, 181 of 324 traumatic deaths (55.9%) occurred in prison, compared with 131 of 503 ACS-related deaths (26.0%). Over the study period, adjusted traumatic mortality rose from 23 to 35 per 100 000 incarcerated individuals, while ACS-related mortality declined from 32 to 20 per 100 000.

**Table 2.** Mechanism and Cause of Deaths

Cause of death	Cases, No. (%)
**Trauma**	
Asphyxiation	451 (63.5)
Blunt	98 (13.8)
Penetrating	38 (5.4)
Suicide^a^	531 (74.8)
Other	123 (17.3)
**Acute care surgery**	
Cirrhosis	571 (68.7)
Gastrointestinal	60 (7.2)
Aneurysm	44 (5.3)
Organ space bleeding	28 (3.4)
Perforated viscus	26 (3.1)
Postoperative complication	18 (2.4)
Abdominal sepsis	16 (1.9)
Hernia	12 (1.4)
SBO	11 (1.3)
Bowel ischemia	8 (1.0)
Pancreatitis	8 (1.0)
Acute cholecystitis	5 (0.6)
Colitis	5 (0.6)
Complicated PUD	5 (0.6)
Volvulus	4 (0.5)
IBD	3 (0.4)
Other	3 (0.4)
Appendicitis	2 (0.2)
Foreign body ingestion	2 (0.2)

Abbreviations: IBD, inflammatory bowel disease; PUD, peptic ulcer disease; SBO, small bowel obstruction.

^a^ Attributed as the cause of death in the database, 444 deaths were due to hanging.

## Discussion

In this large, multistate epidemiological study of 8950 incarcerated individuals who died between 2015 and 2020, nearly 1 in 6 deaths were attributable to trauma or ACS pathologies, with a substantial proportion occurring within prison facilities. These findings suggest potential gaps in access to timely and appropriate medical and surgical care. A Department of Justice report identified operational failures, suicide prevention policy violations, and inadequate medical care as factors contributing to nearly half of federal prison suicides.^6^ These systemic shortcomings likely extend to other medical emergencies, including surgical conditions.

A limitation of this study is the lack of granular clinical information, which restricts our ability to assess whether treatment was received prior to death. Consequently, the location of death may not reliably indicate access to care. Additionally, the lack of incidence data for traumatic and ACS-associated pathologies prevents calculation of case fatality rates. These findings highlight the need to strengthen health care delivery systems in correctional settings and to develop innovative methods to evaluate premortality care access, timeliness, and violence prevention strategies within US prisons.

## Notes

Supplement.
Data Sharing Statement
