# Supplementary material for: Burden of Acute Care Surgery and Trauma-Related Mortality in the US State Prisons
Source: JAMA Netw Open. 2025 Sep 12;8(9):e2531785. doi: 10.1001/jamanetworkopen.2025.31785 (PMC12432632; doi:10.1001/jamanetworkopen.2025.31785)
Supplement: Supplement. — Data Sharing Statement [file jamanetwopen-e2531785-s001.pdf]

Data Sharing Statement

Data
Data available: No

Additional Information
Explanation for why data not available: Data is publicly available at: https://uclacovidbehindbars.org/
